# Experimental investigation on water adsorption and desorption isotherms of the Longmaxi shale in the Sichuan Basin, China

**DOI:** 10.1038/s41598-020-70222-8

**Published:** 2020-08-10

**Authors:** Xinhua Ma, Weijun Shen, Xizhe Li, Yong Hu, Xiaohua Liu, Xiaobing Lu

**Affiliations:** 1 Research Institute of Petroleum Exploration & Development, PetroChina, Beijing, 10083 China; 2 Key Laboratory for Mechanics in Fluid Solid Coupling Systems, Institute of Mechanics, Chinese Academy of Sciences, Beijing, 100190 China; 3 School of Engineering Science, University of Chinese Academy of Sciences, Beijing, 100049 China; 4 State Key Laboratory of Oil and Gas Reservoir Geology and Exploitation, Southwest Petroleum University, Chengdu, 610500 China

**Keywords:** Fossil fuels, Hydrology

## Abstract

The understanding of water adsorption and desorption behavior in the shale rocks is of great significance in the reserve estimation, wellbore stability and hydrocarbon extraction in the shale gas reservoirs. However, the water sorption behavior in the shales remains unclear. In this study, water vapor adsorption/desorption isotherms of the Longmaxi shale in the Sichuan Basin, China were conducted at various temperatures (30 °C, 60 °C) and a relative pressure up to 0.97 to understand the water sorption behavior. Then the effects of temperature and shale properties were analyzed, and the water adsorption, hysteresis, saturation and capillary pressure were discussed. The results indicate that water adsorption isotherms of the Longmaxi shale exhibit the type II characteristics. The water molecules initially adsorb on the shale particle/pore surfaces at low relative pressure while the capillary condensation dominates at high relative pressure. Temperature favors the water sorption in the shales at high relative pressure, and the GAB isotherm model is found to be suitable for describe the water adsorption/desorption behavior. The high organic carbon and full bedding are beneficial to water adsorption in the shales while the calcite inhibits the behavior. There exists the hysteresis between water adsorption and desorption at the whole relative pressure, which suggests that the depletion of condensed water from smaller capillary pores is more difficult than that from larger pores, and the chemical interaction contributes to the hysteresis loop for water sorption. The capillary pressure in the shales can be up to the order of several hundreds of MPa, and thus the desorption of water from the shales may not be as easy as the water adsorption due to the high capillary pressure, which results in water retention behavior in the shale gas reservoirs. These results can provide insights into a better understanding of water sorption behavior in the shale so as to optimize extraction conditions and predict gas productivity in the shale gas reservoirs.

## Introduction

As an available source of natural gas in the world, shale gas has gained extensive attention and has gradually played a significant role in satisfying the growing energy demand^1–3^. Due to the extremely low permeability of shale rocks, the technologies of horizontal drilling and multistage hydraulic fracturing are realized to improve gas production by enhancing the permeability of shale gas formations^4,5^. In the last few years, with the rapid development of horizontal drilling and multistage fracturing technology, the production of shale gas in the United States, Canada and China has increased drastically^6^. According to a survey from the U.S. Energy Information Administration, the global remaining technically recoverable resource of shale gas was estimated as 213 × 10^12^ m^3^ and the proportion of gas production in the United States from shale reservoirs was equal to about 75% in 2019^7^.

Shale gas is an unconventional natural gas, which may exist in one or more of the three forms: free gas, adsorbed gas and dissolved gas, and gas is particularly difficult to extract in that it is adsorbed on the shale and trapped in pores^8–10^. Compared with conventional gas reservoirs, shale rocks are characterized by low porosity, ultra-low permeability and high clay content, such that they have to be commercialized by horizontal drilling and multistage fracturing^6,10^. During the hydraulic fracturing stages, a large variety of water-based fracturing fluids are injected into the shale reservoirs with low concentrations of proppant to create a fracture network to enhance the reservoir permeability. The majority of the injected water is retained in the subsurface with only around 6 ~ 10% recovery of injected water in the shale gas reservoirs in United States^11–13^. Once the fracturing water is absorbed into the shale matrix, the clay minerals will experience swelling, shrinkage and hydration damage. The remaining water-based fluid reduces the relative permeability of the gas phase and affects the effective development of shale gas reservoirs^14–16^. The mechanism of water vapor adsorption and desorption from shale is useful for the fundamental understanding the characterization of the effect of water molecules on methane adsorption behavior, the main content of shale gas. Moreover, the information of water adsorption can provide the relationship between pore-bound water saturation, water vapor pressure and capillary pressure. Therefore, understanding the adsorption/desorption behavior of water vapor in shale rocks is essential to predict the water lock problem in the development of shale gas reservoirs so as to provide the design for the hydraulic fracturing and fracturing fluid.

Water vapor adsorption behavior in shales and other reservoir rocks have been studied for a number of years in mining and geo-energy fields. Chenevert^17^ considered that the strength degradation effect due to water vapor adsorption in shale rocks would cause shale failure, and the migration of water and ions into the shale could alter the petrophysical properties, mechanical strength of shale rocks. Water adsorption by clay-rich shales would result in spalling and induce micro-fractures, and reduce the mechanical strength of shale rocks^18^. Lyu et al.^19^ analyzed shale swelling caused by the water adsorption process, and pointed out that maximum swelling rate occurs at shale's water content of 14%. In order to understand the effect of water distribution on methane adsorption, Li et al.^20^ investigated water adsorption isotherms and concluded that water uptake of clay-rich shales would reduce the methane adsorption capacity. Dutta^21^, Rutqvist^22^ and Zhang et al.^23^ suggested that the retention of fracturing fluid in shale due to water adsorption can cause the expansion and dispersion effect of clay, and the capillary pressure in the pore throat was very large, which was unfavorable to the production of shale gas. Even though there are a few studies on the water adsorption behavior of shales, the characteristics of water adsorption and desorption on the shale reservoir rocks are still not fully understood. Consequently, it is extremely necessary to understand water sorption behavior of shales so as to optimize extraction conditions and ultimately maximize gas production in shale gas reservoirs.

In this study, water vapor adsorption and desorption isotherms of the Lower Silurian Longmaxi shale samples from the Sichuan Basin, China were measured using thermal gravimetric sorption analysis at different temperatures and a relative vapor pressure up to 0.97. The water vapor adsorption behavior in the shales was analyzed, and the effects of temperature and shale properties were evaluated. Then the hysteresis and capillary condensation were discussed, and the GAB and FHH isothermal models were applied to describe the water adsorption/desorption behavior. Furthermore, the relationship between water adsorption, saturation and capillary pressure were determined to explain the absorption-imbibition and drainage-desorption process in shale gas reservoirs.

## Experimental Materials and methods

### Shale samples, preparation and characterization

**Figure 1:**
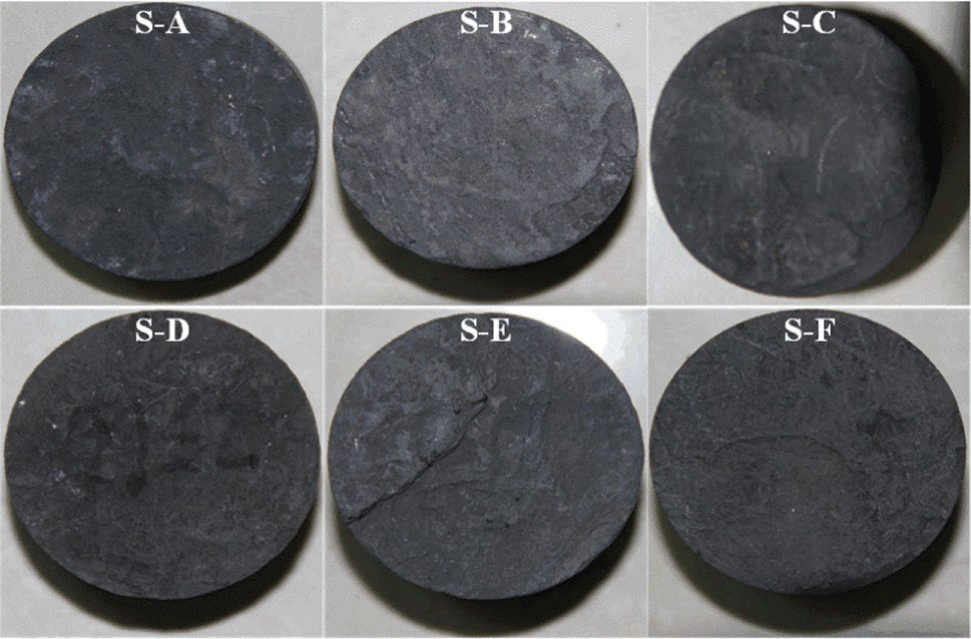
Shale samples from various shale wells within the Lower Silurian Longmaxi Formation.

A total of six different shale samples used in this study were collected from various shale wells within the Lower Silurian Longmaxi Formation in the Sichuan Basin, China, which ranges from 2,517.63 m to 2,570.00 m, and these shale samples are illustrated in Fig. 1. The shale samples were first crushed and sieved to different particles, whose size range from 250 μm to 800 μm before the measurement. Parts of particles were used for the water sorption measurement, and others were used for shale characterization. Total organic carbon (TOC) was measured at 1,000 °C by using a Multi N/C 3,100 TOC-TN analyzer. The mineral composition was measured by using the XRD Smartlab 9Kw analyzer. The specific surface area (SSA) of shale samples was obtained from N_2_ adsorption isotherms at 77 K using Micromeritics ASAP2420 adsorption apparatus. And the characteristics of each shale sample are summarized in Tables 1 and 2.

**Table 1:** Some measured characteristics of shale samples used in this study.

No.	TOC (%)	Density (g/cm^3^)	Porosity (%)	Specific surface area (m^2^/g)	Bedding
S-A	1.17	2.36	5.84	9.60	Full
S-B	1.30	2.32	6.22	5.23	Full
S-C	5.83	2.28	6.85	28.24	Full
S-D	1.06	2.54	5.15	8.96	Less
S-E	1.90	2.35	5.52	17.62	Less
S-F	2.70	2.30	6.45	17.51	Less

**Table 2:** Major mineralogical composition of shale samples based on XRD analysis (%).

No.	Quartz	Feldspar	Calcite	Dolomite	Kaolinite	Pyrite
S-A	38.3	37.0	7.1	14.6	–	3.0
S-B	38.8	58.7	–	–	–	2.5
S-C	35.9	62.1	–	–	–	2.0
S-D	34.5	–	49.2	13.6	–	2.7
S-E	38.8	50.9	–	–	8.5	1.8
S-F	40.4	58.1	–	–	–	1.5

In this study, six shale samples with the particle size range of 500–800 µm were selected and placed in 6 jars, and their weights were measured using a balance. Then, each shale sample was placed in a drying oven at 120 °C for 24 h. After drying, each sample was placed on the desiccator with silica gel and allowed cooled to room temperature, and these samples were weighed and then returned to the desiccator. Next, an incubator was prepared and the intactness was checked. The temperature was adjusted to 30 °C, and the temperature deviation was not allowed to exceed ± 0.1 °C prior to the following experiment. Subsequently, various saturated salt solutions to be used in the experiment were prepared, and the saturated salt solutions under different humidity conditions are shown in Table 3^24^.

**Table 3:** Saturated salt solutions used to control relative humidity.

Salt solution	Relative humidity (%)	
	30 °C	60 °C
LiCl	11.28	10.95
MgCl_2_	32.44	29.26
NaBr	56.03	49.66
KI	67.89	63.11
NaCl	75.09	74.50
KCl	83.62	80.25
K_2_SO_4_	97.00	94.56

### Experimental methods

**Figure 2:**
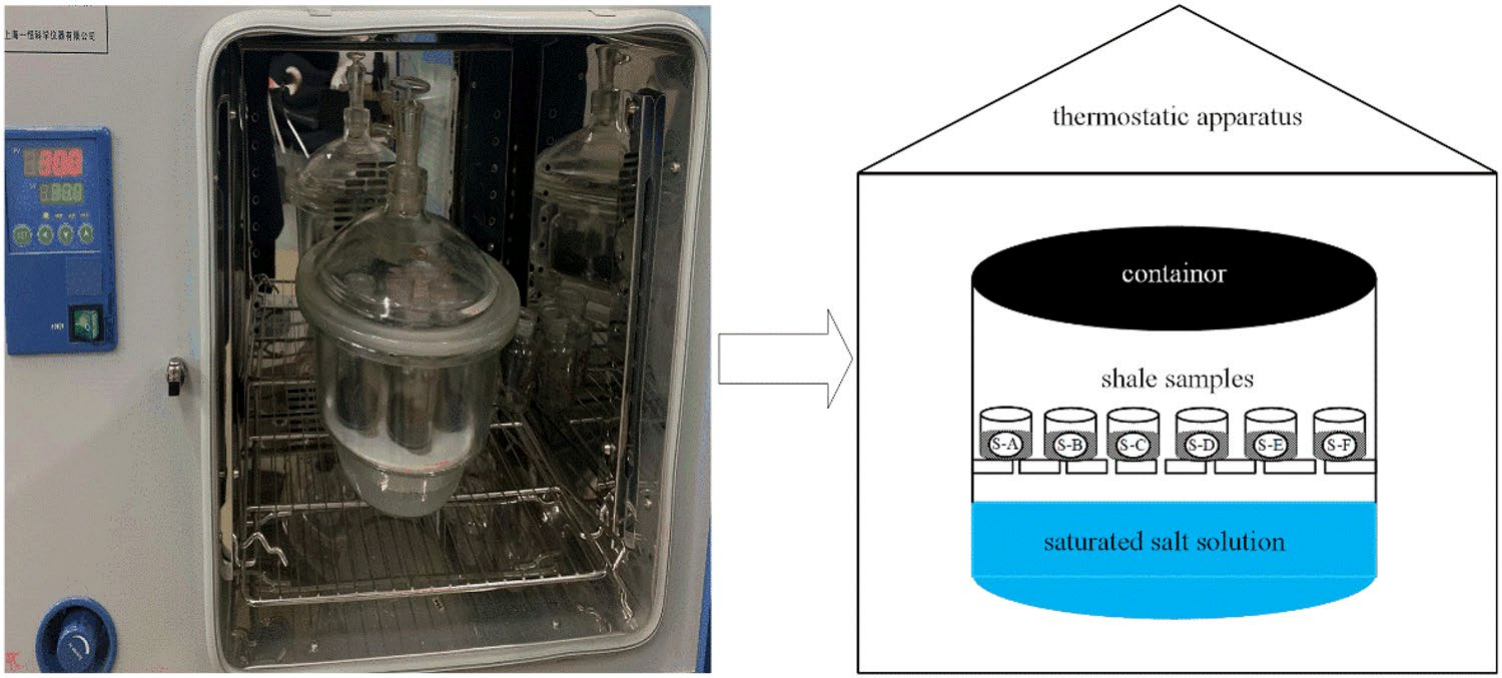
Schematic apparatus used to measure water adsorption and desorption isotherms.

The experimental principle of water vapor adsorption and desorption of shale is mainly based on the equilibrium relationship between the water content in shale and the external water content. The dry shale samples were placed in an environment with a constant relative humidity controlled by a saturated salt solution. Relative humidity is the percentage of water vapor pressure in the air (*p*) relative to the saturated water vapor pressure (*p*_*0*_) at a given temperature, which is known as relative pressure (*p/p*_*0*_)^25^. When the water content in shale is less than the humidity in the environment, water vapor will diffuse and adsorb onto the shale, and the water content of the shale will increase. When the water content of shale is greater than the external humidity, the water vapor can desorb and diffuse out of the shale, reducing the water content of the shale. The change in shale mass during this period represents the amount of water vapor adsorbed (desorbed) by (from) the shale. The experimental apparatus used for measuring shale water vapor adsorption and desorption were shown in Fig. 2.

In this study, the jars (corks opened) containing each shale were placed in a desiccated container with a saturated LiCl salt solution on the bottom and then placed in a 30 °C incubator, as shown in Fig. 2. After a certain period of time, the jars were taken out and weighed once per day. The water vapor adsorbed by shale was considered to be at equilibrium when the weight of several consecutive measurements was unchanged (7 ~ 15 d was usually required to achieve equilibrium). Then, the jars containing shale samples were removed from the desiccator, placed in a drying cabinet (120 °C, 24 h), dried, weighed, and placed in a saturated MgCl_2_ salt solution for the next set of adsorption experiments. The experimental procedure was the same as that for the saturated LiCl salt solution, and the isothermal adsorption experiments of NaBr, KI, NaCl, KCl and K_2_SO_4_ were followed. When the water vapor isothermal adsorption experiment of shale at 30 °C was completed, and the isothermal desorption experiment at 30 °C was carried out. The shale-filled jars were removed from the desiccated container with the saturated K_2_SO_4_ salt solution on the bottom and placed in desiccated container with a saturated KCl salt solution on the bottom and then placed in a 30 °C incubator for desorption experiments. The jars were removed once per day for weighing purposes after a certain period of time. When subsequent weighing results no longer changed, the water vapor desorption of shale was considered to reach an equilibrium state. Then the saturated NaCl, KI, NaBr, MgCl_2_ and LiCl salt solutions were sequentially used for desorption experiments. And the experiments of water adsorption and desorption isotherm of shale at 60 °C were the same as that at 30 °C.

## Isothermal adsorption model

The model of adsorption isotherms is a useful approach to describe the equilibrium phenomenon between the adsorbed amount and the pressure of the adsorbate at a constant temperature, which provided the valuable information on pore structure and capacity in porous materials^26–28^. In order to describe and analyze the sorption phenomenon, a number of isothermal models such as Langmuir, Freundlich, Brunauer–Emmett–Teller (BET), Guggenheim-Anderson-de Boer (GAB), and Frenkel-Halsey-Hill (FHH) models have been proposed over the years^29–31^. These mathematical models are usually used for describing and predicting the isothermal adsorption process, including adsorption equilibrium and isotherm parameters. In this study, some common isotherm models were used to fit the adsorption and desorption isotherms and to understand the adsorption process in shale reservoir rocks.

### BET model

A common isotherm model describing the adsorption process of a gas on reactive surfaces is the BET isotherm model. The BET isotherm, assuming Langmuir adsorption at low surface coverage, has been proposed to account for multilayer adsorption, particularly for Type II isotherm characteristics, which was widely used to determine the specific surface area of adsorbents in the relative pressure range between 0.05 and 0.3^32,33^. The already adsorbed molecules provide new sites for added molecules resulting in the formation of at least a second layer of adsorbed molecules. The molecules adsorbed at the solid surface are assumed to condensate as a liquid phase. The BET model normally predicts too little adsorption at low pressures and too much adsorption at high pressures, which can be written as

1 $$q = \frac{{\mathop q\nolimits_{m} \mathop c\nolimits_{B} \mathop a\nolimits_{w} }}{{(1 - \mathop a\nolimits_{w} )(1 - \mathop a\nolimits_{w} \mathop { + c}\nolimits_{B} \mathop a\nolimits_{w} )}}$$

where $q$ is the amount of gas adsorption, $\mathop q\nolimits_{m}$ is the maximum amount of adsorption for the monolayer, $\mathop a\nolimits_{w}$ is the water activity, $\mathop a\nolimits_{w} = {p \mathord{\left/ {\vphantom {p {\mathop p\nolimits_{0} }}} \right. \kern-\nulldelimiterspace} {\mathop p\nolimits_{0} }}$, $\mathop c\nolimits_{B}$ is the BET constants, which are related to the single-layer and multi-layer adsorption properties, respectively.

### GAB model

Based on the theory of multi-layer adsorption, the GAB isothermal adsorption model was developed by Guggenheim^34^, Anderson^35^ and Boer^36^ to describe monolayer and multilayer adsorption. The GAB isotherm is a modification of the Langmuir and BET theories of adsorption isotherms, which assumes that the water adsorption is a multilayer phenomenon without lateral interactions of water molecules. And the isotherm model introduces an additional parameter *k*_*G*_, which is related to the multi-layer adsorption properties. The GAB isothermal model can be expressed as

2 $$q = \frac{{\mathop q\nolimits_{m} \mathop c\nolimits_{G} \mathop k\nolimits_{G} \mathop a\nolimits_{w} }}{{(1 - \mathop k\nolimits_{G} \mathop a\nolimits_{w} )(1 - \mathop k\nolimits_{G} \mathop a\nolimits_{w} \mathop { + c}\nolimits_{G} \mathop k\nolimits_{G} \mathop a\nolimits_{w} )}}$$

where $\mathop c\nolimits_{G}$ and $\mathop k\nolimits_{G}$ are the GAB constants, respectively, the remaining parameters are the same as above. Noted that the GAB equation resembles the BET model when $\mathop k\nolimits_{G}$ is equal to 1, and it can be regarded as the addition of the Langmuir monolayer adsorption and the multilayer adsorption corresponding to Raoult's law^17^. It was indicated that the GAB model was applicable for a wide range of water activity from 0.1 to 0.9 for different clay types^33^.

### FHH model

Based on the idea of a potential field caused by the adsorbent surface that acts on the adsorbate molecules, the FHH isotherm model of fractal interface was proposed by Frenkel^37^, Halsey^38^ and Hill^39^. Considering the effect of the replacement of the solid by the liquid, the FHH isotherm assumes the adsorbate as a uniform thin layer of liquid on a planar, homogeneous, solid surface. The FHH model generally fits adsorption isotherms over the range of relative pressure from 0.1 to 0.8, which is described as

3 $$\left( {\frac{q}{{q_{m} }}} \right)^{n} = \frac{A}{{ - \ln (p/p_{0} )}}$$

4 $$A = \frac{{\upvarepsilon _{0} }}{{RTx_{m}^{n} }}$$

where *n* is an empirical parameter, which is related to the adsorbate, adsorbent and temperature; $\mathop \varepsilon \nolimits_{0}$ is the potential of the solid surface for adsorption; $\mathop x\nolimits_{m}^{n}$ is the monolayer adsorption thickness; *T* is the temperature; *R* is the gas constant; and the remaining parameters are the same as above.

## Results and discussion

### Water vapor adsorption isotherm

**Figure 3:**
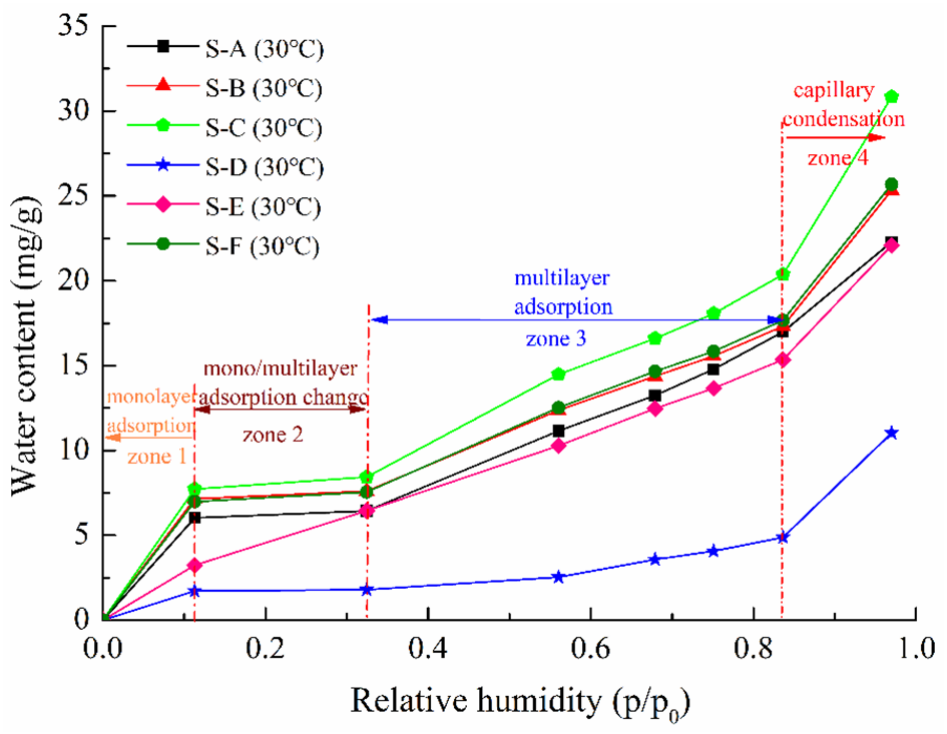
Water vapor adsorption isotherm for shale samples at 30 °C.

The water vapor adsorption isotherms of all six shale samples at the temperature of 30 °C are shown in Fig. 3. According to the standard classification of the International Union of Pure and Applied Chemistry (IUPAC)^40^, the water vapor sorption behavior of these shale samples exhibits type II shape. As can be seen from the Fig. 3, the water vapor isothermal adsorption curves of these shale samples have similar variation characteristics except for the shale S-E. The water adsorption isotherms can be roughly classified into four zones: monolayer adsorption, mono/multilayer adsorption change, multilayer adsorption and capillary condensation. At low relative pressure (0–0.11), there is a rapid increase in water adsorption where monolayer adsorption dominates, and then a slow increase follows over the range of 0.11 to 0.32 where multilayer sorption arises after monolayer adsorption ends. With the increasing relative pressure (0.32–0.83), a moderate increase in water adsorption occurs where multilayer sorption dominates, and finally there is a rapid increase at high relative pressure (0.83–0.97) where capillary condensation is the dominant process. However, there is only one inflection point (*p/p*_*0*_ ~ 0.83) for the shale S-E with the kaolinite, and the adsorption isotherm is separated into two zones: a rapid increase at relative pressure (< 0.83) followed by a linear high-gradient zone from 0.83 to 0.97. At relative pressure below 0.83, the water adsorption isotherm increases approximately linearly because of the single-molecule and multi-molecular layer adsorption of water vapor to the shale surface. As the relative pressure continues to increase, the water content increases sharply and the change is an indication that capillary condensation has taken place in the shale pores, which causes a sharp increase in water content. Several studies have observed that the water adsorption isotherm for kaolinite with linear form at high relative pressure^41,42^.

### Effect of temperatures on water vapor adsorption

**Figure 4:**
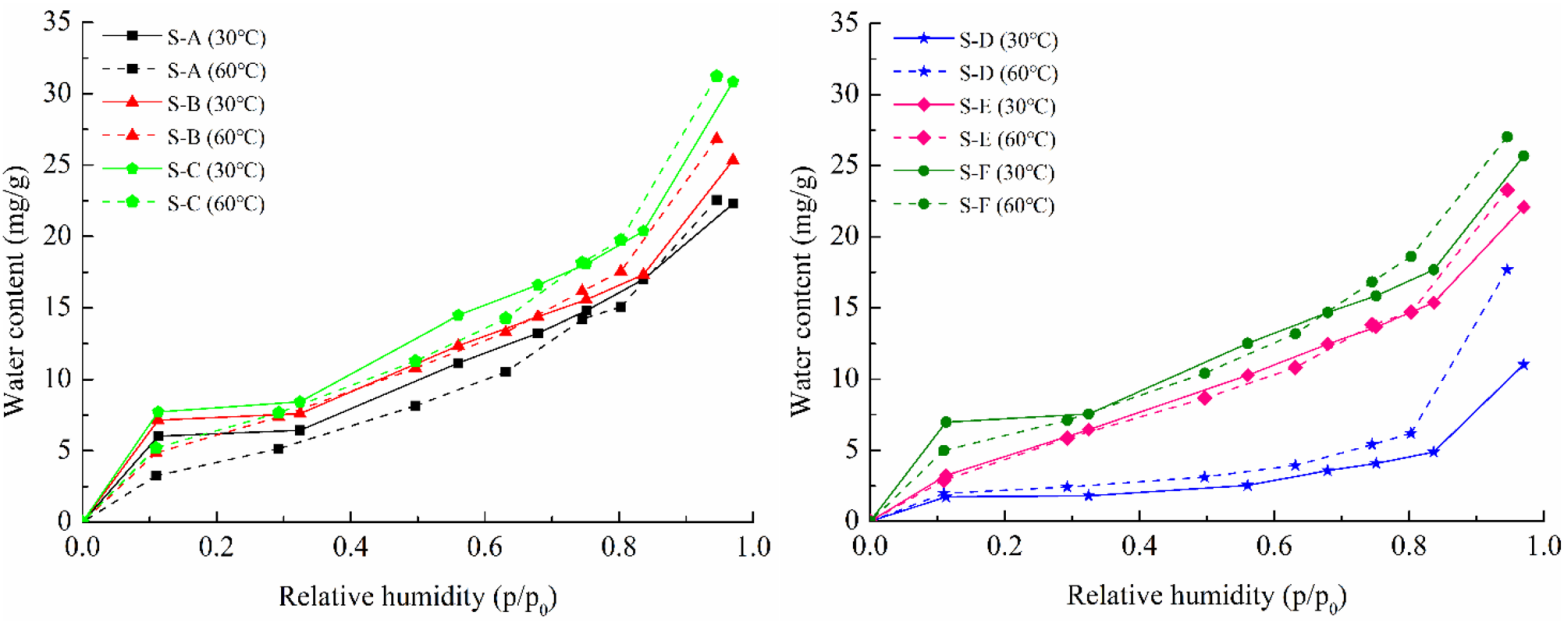
Effect of temperatures on water vapor adsorption for shale samples.

The water vapor isothermal adsorption curves of different shale samples at 30 °C and 60 °C with a relative pressure up to 0.97 are illustrated in Fig. 4. As can be seen from the Fig. 4, the effect of temperatures on the amount of water vapor adsorbed by each shale sample varies similarly. At low relative pressure, the changes in the amount of water adsorbed with temperature are small. With increasing relative pressure, the increase of water adsorbed was more pronounced at high temperature. This may be resulting from the temperature-dependent chemical adsorption or the presence of solutes in the adsorbate. Due to the energy requirement of the chemical adsorption, the extent to which this behavior occurs increases with temperature, and consequently the total amount of water adsorbed on the shale rocks increases at high relative pressure. It has been mentioned that the weak chemisorption of water on cement pastes arose at room temperature^43^. As a consequence, the chemical interaction of water molecules with the shale surface is inevitably present in this process. The other reason is that there exists the salt component in the shale reservoir rocks. In the water vapor-dominated reservoirs, the salinity of reservoir rocks has long been considered as an important reason for the decrease of vapor pressure^44,45^. The minerals in the reservoir rocks will dissolve in the adsorbed water and thus have an appreciable effect on the adsorption. As temperature increases, the solubility of the salts in the formation increases, which influences water vapor pressure. And this may in turn affect the amount of water adsorbed by shale at high relative pressure.

### Effect of shale properties on water vapor adsorption

**Figure 5:**
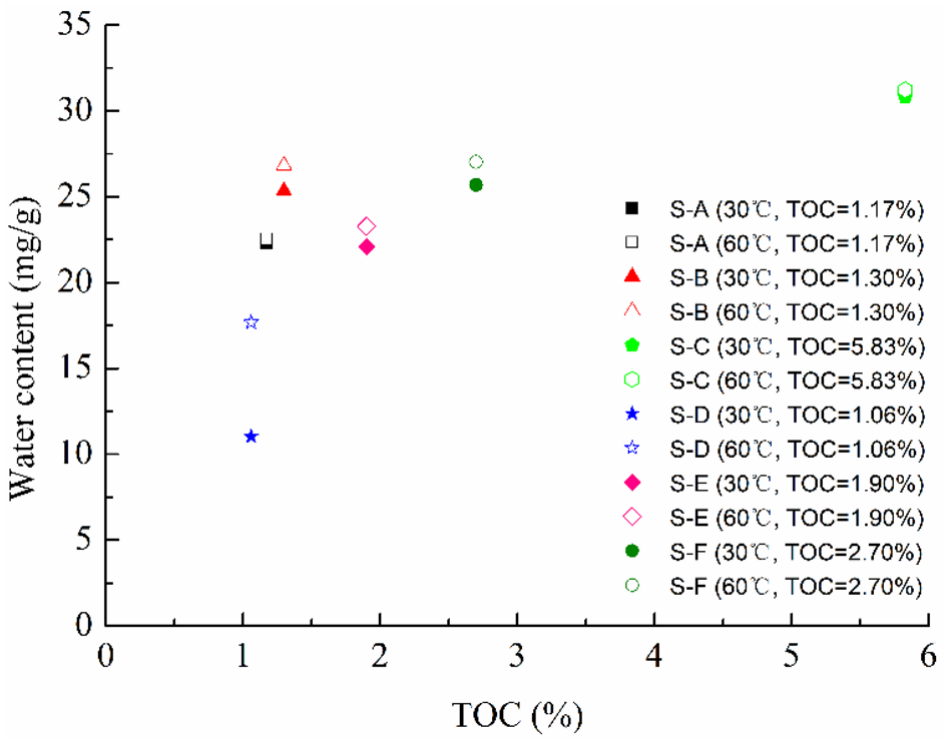
Effect of TOC on water vapor adsorption for shale samples.

The water adsorption isotherms are dependent on the rock properties, which is resulting from the specific variations of mineralogical composition and physicochemical structure^42,45^. The behavior is more complicated due to the presence of hydrophobic/hydrophilic organic kerogen and hydrophilic clay minerals. The relationship between the water adsorption capacity and the TOC content of all six shale samples at 30 °C and 60 °C is presented in Fig. 5. As can be seen from the Fig. 5, the higher the organic carbon content of shale with the same bedding is, the greater the adsorption capacity is, which indicates that the organic carbon content plays an active role in water vapor adsorption of shale reservoir rocks. Organic matter in shale contains many nano-pores, and the organic carbon content affects the water adsorption in shale reservoir rocks. The result is in accordance with the previous studies that the organic kerogen favors the adsorption capacity^13,46^. Compared with these shale samples, the shale samples with full bedding are the higher adsorption than those with less bedding, and the shale bedding will impact on shale reservoir properties and drilling optimization^44^. Among the shale S-B, S-C and S-F, the shale S-C exhibits the highest water adsorption while the shale S-B shows the lowest water adsorption. This is consistent with the result that relatively high SSA tends to have a higher water adsorption capacity for the shales without regard for the expansive clays. The X-ray diffraction analyses show that quartz and pyrite were common in these three shale samples. Moreover, it is notable that the shale S-A and S-D exhibit the low adsorption capacity than other shale samples, and the behavior is in accord with that the calcite detected in them. The calcite is characterized by high surface area and weak affinity for water reported in the previous studies^47,48^, and the reservoir rocks with calcite will display the relatively lower water adsorption capacity. Therefore, the water adsorption behavior in the shales are very closely related with the mineralogy and physicochemical structure and are more complex compared with conventional reservoir rocks.

### Hysteresis behavior and capillary condensation

**Figure 6:**
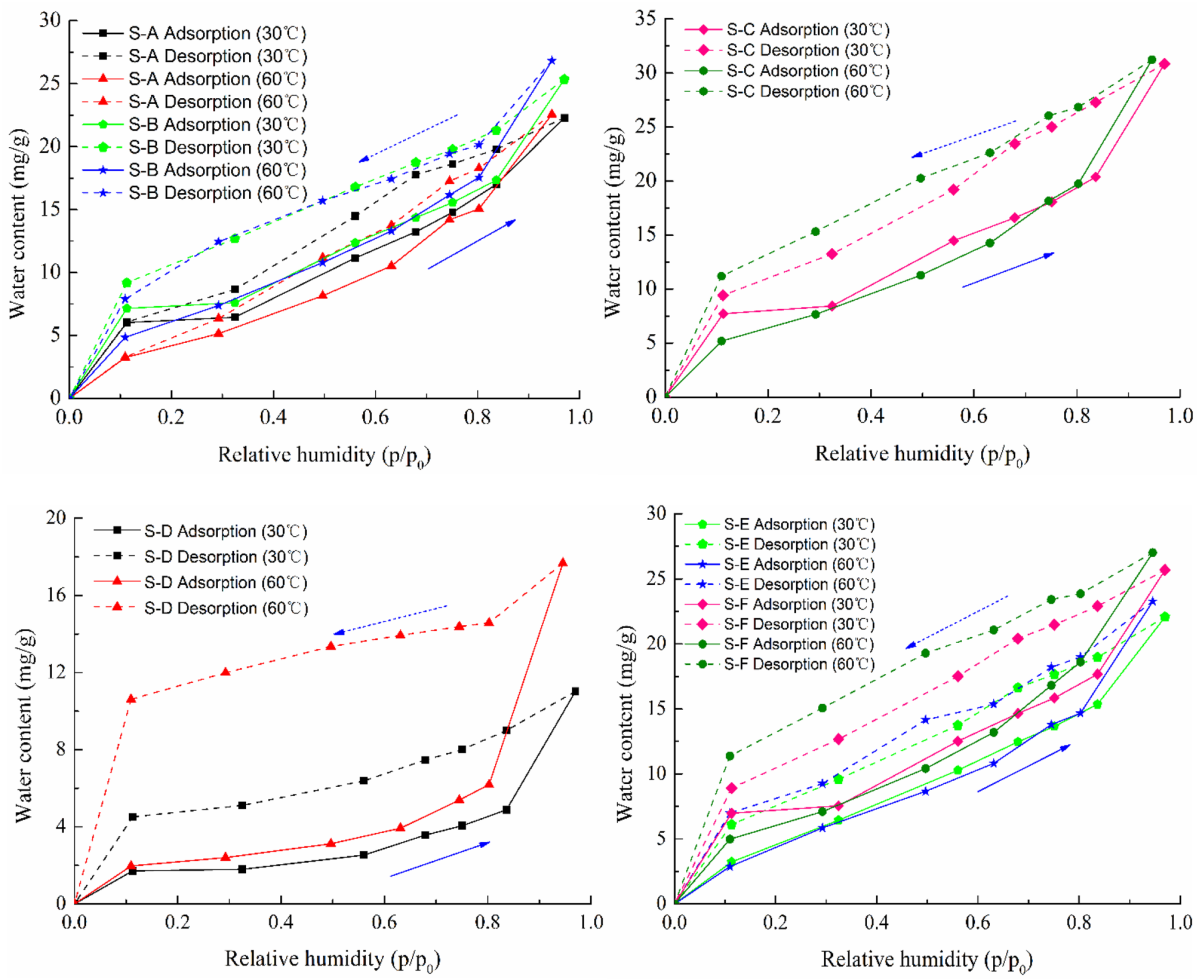
Water vapor adsorption–desorption isotherms for shale samples.

The water vapor adsorption–desorption isotherms of different shale samples at 30 °C and 60 °C are illustrated in Fig. 6. As can be seen from the Fig. 6, the water vapor adsorption and desorption curves of all shale samples do not coincide, and there exists the hysteresis behavior in the whole relative pressure. The monolayer adsorption occurs and dominates at low relative pressure, and the pore space will be filled up before multilayer adsorption is developed. As relative pressure increases, the multilayers of adsorbed water form in the larger pores. At a high relative pressure, the capillary condensation commences in the small pores due to the ink-bottle effect^49^. The desorption curve is above the adsorption curve, and the desorption of water from the shale pores is more difficult than the adsorption of water by the shale due to the capillary forces. The adsorption hysteresis appears in the interval, and the desorption amount of water vapor is far less than the adsorption amount. This implies that the water retention by capillary condensation is not easily desorbed from shale as relative pressure decreases, which explains the small amount of fracturing fluid returned to the ground after hydraulic fracturing in the shale gas reservoirs and that most of the fracturing fluid remains in the shale formations, which affects the effective development of the shale gas reservoirs^12,19^. Moreover, the hysteresis loop exists in the whole relative pressure, and the behavior indicates that the irreversible change may occur in the pore structure on water adsorption. As discussed above, the chemical interaction of water molecules with the shale surface emerges during the water adsorption.

### Adsorption and desorption isotherm fitting

**Figure 7:**
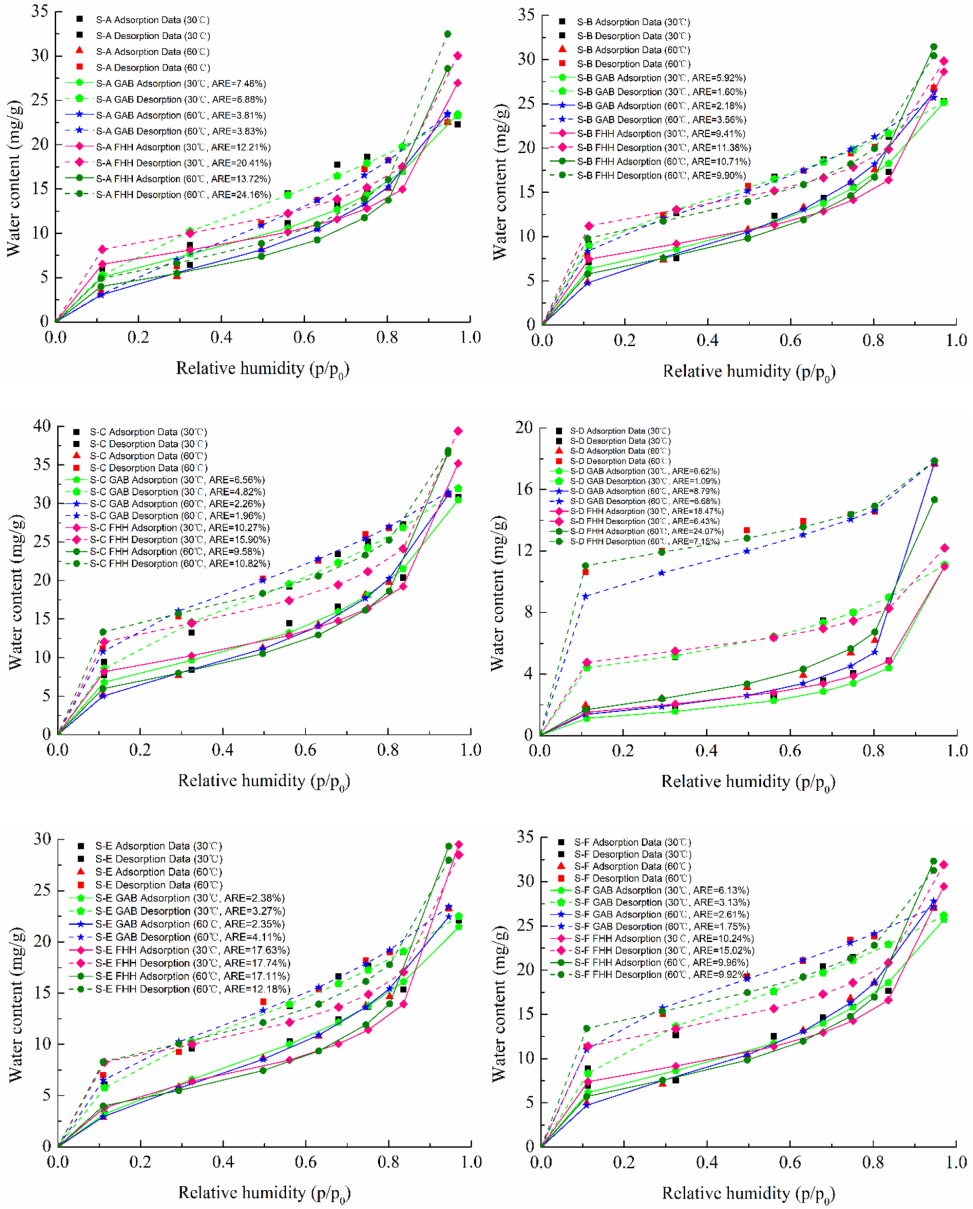
Measured and fitted sorption isotherms for shale samples.

The mathematical models of water sorption isotherms are significant for analyzing the water sorption mechanisms and distribution characteristics in porous materials^30^. Based on the experimental adsorption and desorption measurements of all six shale samples at 30 °C and 60 °C, the isothermal adsorption models of GAB and FHH were used to fit the isotherms data, which is illustrated in Fig. 7. As can be seen from the Fig. 7, the fitting results of GAB model are good in general. The average relative error (ARE) in which is less than that of FHH model, and it provides a tendency to estimate the experimental data, which minimizes the error distribution across the entire range. The GAB isotherm is an extension of the BET isotherm, and it is the most common model used to determine the sorption isotherms, which was suitable for a wide range of relative pressure for different clay types^33^. However, as shown in Fig. 7, the FHH isotherm model deviates greatly from the fitting results of the shale samples at the high relative pressure, even though some previous studies have reported that the FHH isothermal model was widely used to describe the phenomenon of capillary condensation of porous materials at high relative pressure^39,50^. Therefore, the GAB isothermal model is optimal to fit the water adsorption and desorption isotherms of the shale samples, which can be used to describe the water sorption isotherm in the shale.

### Water adsorption and water saturation

The water content of the rock is the quantity of water contained in the rock pores, which can be described as the volumetric water content and weight water content, respectively^51,52^. It can be expressed as

5 $$\theta = \frac{{V_{W} }}{{V_{T} }}$$

6 $$W_{g} = \frac{{M_{W} }}{{M_{T} }} = \frac{{\rho_{W} V_{W} }}{{\rho_{T} (1 - \varphi )V_{T} }}$$

where $\theta$ is the volumetric water content of the rock; $\mathop V\nolimits_{W}$ is the water volume of the rock; $\mathop V\nolimits_{T}$ is the total volume of the rock; $\mathop W\nolimits_{g}$ is the water content of the unit mass of the rock; $\mathop M\nolimits_{W}$ is the water mass of the rock; $\mathop M\nolimits_{T}$ is the mass of dry rock; $\mathop \rho \nolimits_{W}$ is the water density; $\mathop \rho \nolimits_{T}$ is the rock density; and $\varphi$ is the rock porosity.

Water saturation of the rock is the ratio of the pore volume occupied by water in a rock to the pore volume of the rock^53^, and it may be expressed as

7 $$S_{W} = \frac{{V_{W} }}{{V_{V} }} = \frac{{V_{W} }}{{{\varphi }V_{T} }}$$

By combining the Eq. (5), (6) and (7), water saturation can be written as

8 $$S_{W} = \frac{(1 - \varphi )}{\varphi }\frac{{\rho_{T} }}{{\rho_{W} }}W_{g}$$

**Figure 8:**
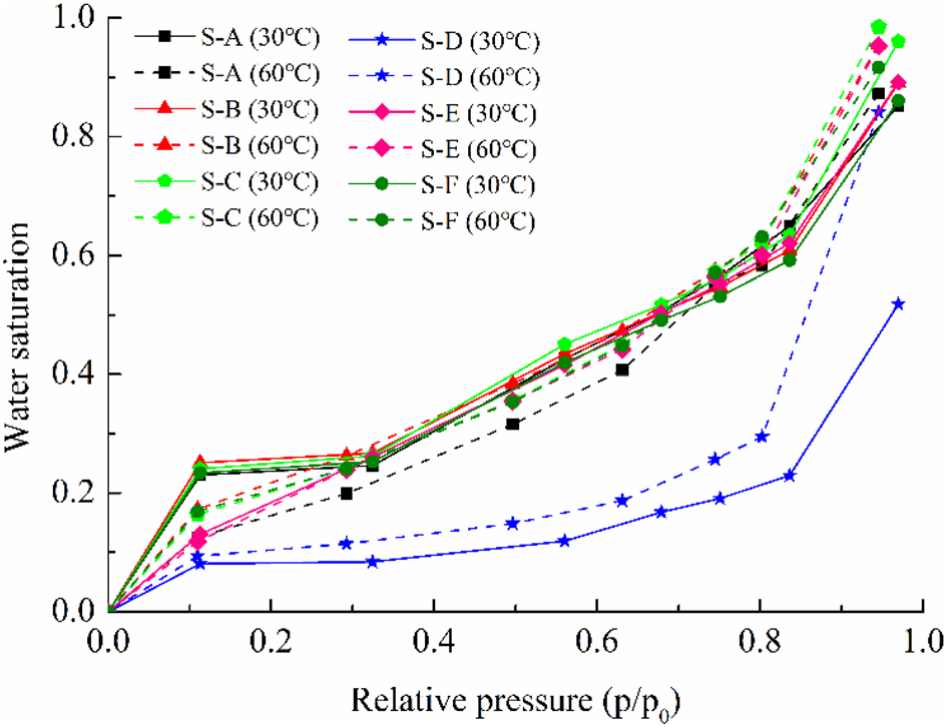
Relative pressure versus water saturation for shale samples.

Based on the above Eq. (8), the amount of water adsorption at any given pressure and temperature can be turned into water saturation, which is shown in Fig. 8. The water saturation levels are calculated from the amount of adsorbed water using the water density of 0.9956 g/cm^3^ (0.9983 g/cm^3^) at 30 °C (60 °C), and the porosity and density of the shales are presented in Table 1, respectively. As can be seen from the Fig. 8, the water saturation in shale is low at relative pressures below 0.8, and the water adsorbed exists in the pore space as surface water, where the adsorption dominates as mentioned above. When the relative pressure exceeds 0.8, water saturation increases sharply, which indicates that capillary condensation is a dominant process at high relative pressure. Consequently, the increase in relative pressure will undoubtedly lead to a more dramatic increase in water saturation.

### Water saturation and capillary pressure

The adsorption behavior is usually described through the isotherm models while the Kelvin equation can be used to illustrate capillary condensation^54^. And it can be expressed as

9 $$RT\ln (p/p_{0} ) = \frac{{2\upgamma V_{m} }}{r}$$

where *R* is the universal gas constant; *T* is the temperature; $\gamma$ is the interfacial tension; $\mathop V\nolimits_{m}$ is the molar volume of a condensed droplet; $r$ is the radius of the droplet; and the remaining parameters are the same as above.

Combined with the Laplace's Eq. (10), the capillary pressure can be written as

10 $$\mathop P\nolimits_{{\text{c}}} = \frac{2\gamma }{{\text{r}}}$$

11 $$\mathop P\nolimits_{{\text{c}}} = \frac{RT}{{\mathop V\nolimits_{m} }}\ln \left( {{p \mathord{\left/ {\vphantom {p {\mathop p\nolimits_{0} }}} \right. \kern-\nulldelimiterspace} {\mathop p\nolimits_{0} }}} \right)$$

where *P*_*c*_ is the capillary pressure.

**Figure 9:**
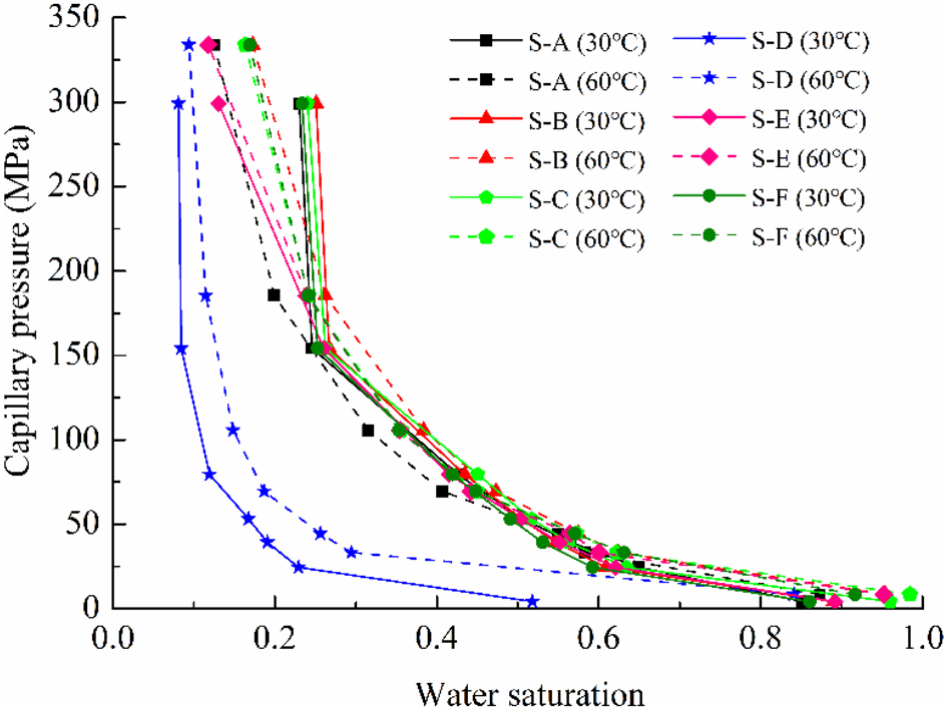
Water saturation versus capillary pressure for shale samples.

According to the Eq. (11), the experimentally measured adsorption isotherms in this study can be turned into the capillary pressure curve, which is illustrated in Fig. 9. As can be seen from the Fig. 9, the capillary pressure can reach the order of several hundreds of MPa in the shale rocks with low permeability, and that is why the desorption of water from the shale may not be easy as the water adsorption, which results in the adsorption/desorption hysteresis. Water retention in the shale formation plays a critical role in fluid trapping due to the high capillary pressure in the two-phase flows in shale gas reservoirs as well as in the saturation of shale formations after the multistage fracturing treatment^12,55^. Thus it is very difficult for fracturing water to return the surface, and there exists the water blocking in the shale gas reservoirs. The shale swelling and shrinkage may be resulting from the changes in the suction or variation in the degree of water saturation. Therefore, there is great significance to understand the water retention behavior in shale gas reservoirs.

## Conclusions

In this study, the measurements of water vapor adsorption/desorption isotherms of the Lower Silurian Longmaxi shale in the Sichuan Basin, China were performed at various temperatures (30 °C, 60 °C) and relative pressures (0–0.97) using the gravimetric method. The effects of temperature and shale properties on water adsorption behavior were analyzed, and then the hysteresis and capillary condensation were discussed. Furthermore, the water adsorption, saturation and capillary pressure were determined to illustrate the water retention in shale gas reservoirs. According to the above results, the following conclusions can be drawn from this study: (1) The water adsorption isotherms of the Longmaxi shale are characterized as type II behavior. The water molecules initially adsorbed on the shale particle/pore surfaces at low relative pressure while the capillary condensation starts to dominate at high relative pressure (*p/p*_*0*_ > 0.83). Temperature has a positive impact on water adsorption uptake in the shales which results in higher adsorbed water at high relative pressure. The GAB model is shown to be suitable for describe the water adsorption/desorption on the shales at the entire relative pressure. (2) The water adsorption behavior is closely related to both mineralogical composition and physicochemical structure. The high organic carbon and full bedding are beneficial to water adsorption on the shales while the calcite inhibit the behavior. (3) There exists the hysteresis loop between water adsorption and desorption isotherms on the Longmaxi shale. The hysteresis behavior is more pronounced at low relative pressure, which implies that the depletion of condensed water from smaller capillary pores is more difficult than that from larger pores. And the chemical interaction of water molecules with the shale surface chemical interaction contributes to the hysteresis loop for water adsorption. (4) The capillary pressure in the shales can be up to the order of several hundreds of MPa in magnitude, and thus the desorption of water from the shales is more difficult than the water adsorption, which causes the adsorption/desorption hysteresis to very low pressure. The water retention behavior in the shale gas reservoirs plays a critical role in water trapping due to the high capillary pressure so as to affect the extraction of shale gas from shale formations.
